# A multicenter retrospective cohort study on predicting the risk for amiodarone pulmonary toxicity

**DOI:** 10.1186/s12890-022-01926-y

**Published:** 2022-04-05

**Authors:** Wang Chun Kwok, Ting Fung Ma, Johnny Wai Man Chan, Herbert H. Pang, James Chung Man Ho

**Affiliations:** 1 Department of Medicine, Queen Mary Hospital, The University of Hong Kong, 4/F, Professorial Block, 102 Pokfulam Road, Hong Kong Special Administrative Region Pokfulam, China; 2 Department of Statistics, University of Wisconsin, Madison, WI USA; 3 Department of Medicine, Queen Elizabeth Hospital, 30 Gascoigne Road, Yau Ma Tei, Hong Kong Special Administrative Region China; 4 Li Ka Shing Faculty of Medicine, School of Public Health, The University of Hong Kong, 7 Sassoon Road, Pokfulam, Hong Kong Special Administrative Region China; 5 Department of Biostatistics and Bioinformatics, School of Medicine, Duke University, Durham, NC USA

**Keywords:** Amiodarone, Pulmonary toxicity, Pneumonitis, Adverse events

## Abstract

**Background:**

Amiodarone is one of the most commonly used anti-arrhythmic agents. Amiodarone pulmonary toxicity is a potentially fatal adverse effect associated with amiodarone use. Previous studies on the epidemiology and risk factors for amiodarone pulmonary toxicity showed diverse results.

**Methods:**

A multicenter retrospective cohort study was conducted to identify clinic-epidemiologic markers associated with amiodarone pulmonary toxicity for development of a prediction rule. Patients taking amiodarone who were managed in 3 centres in Hong Kong from 2005 to 2015 were included in this study. Penalized logistic regression was used to model the outcome as it is rare.

**Results:**

A total of 34 cases with amiodarone pulmonary toxicity were identified among 1786 patients taking amiodarone for at least 90 days from 2005 to 2015. The incidence of amiodarone pulmonary toxicity was estimated to be 1.9%. The risk factors for amiodarone pulmonary toxicity included advanced age (OR 1.047, 95% CI 1.010–1.085, *p* = 0.013), ventricular arrhythmia (OR 2.703, 95% CI 1.053–6.935, *p* = 0.039), underlying lung disease (OR 2.511, 95% CI 1.146–5.501, *p* = 0.021) and cumulative dose of amiodarone (OR 4.762, 95% CI 1.310–17.309 *p* = 0.018).

**Conclusions:**

The incidence of amiodarone pulmonary toxicity in Chinese patients in Hong Kong is estimated to be 1.9% in this study. Age, underlying lung disease, ventricular arrhythmia and cumulative dose of amiodarone are associated with the development of amiodarone pulmonary toxicity. A prediction rule was developed to inform the risk of developing amiodarone pulmonary toxicity.

## Introduction

Amiodarone is a commonly used anti-arrhythmic agent that is lipophilic in nature with large volume of distribution with variable elimination half-life [1]. The prevalence of the adverse side effects associated with amiodarone use was as high as 15% in the first year and 50% with long-term therapy [2]. The important adverse effects of amiodarone include pulmonary toxicity, thyroid dysfunction and hepatotoxicity. According to clinical trials and observational studies, the reported incidence of amiodarone pulmonary toxicity varies from 1 to 10% [3–7]. One of the proposed pathogenic mechanisms for amiodarone pulmonary toxicity is direct pulmonary toxicity from reduced phospholipid degradation and its accumulation which leads to lipid-laden macrophages formation, lipid peroxidation, reactive oxygen radicals generation, disturbance of cellular calcium and prostaglandin metabolism, and deposition of collagens resulting in lung injury [8, 9]. Interstitial pneumonitis is the most common clinical manifestation of amiodarone pulmonary toxicity [10, 11]. Other clinical patterns of amiodarone pulmonary toxicity include eosinophilic pneumonia [12], organizing pneumonia [13], acute respiratory distress syndrome (ARDS) [14], diffuse alveolar haemorrhage [15], lung nodules [16] and pleural effusion [17].

Reported risk factors of amiodarone pulmonary toxicity include age above 60 years old, duration of amiodarone use of 6 to 12 months [18], pre-existing lung disease especially chronic obstructive pulmonary disease (3, 19), male gender, underlying renal disease [3] and elevated plasma monodesethylamiodarone concentration (4). Despite the fact that majority of amiodarone pulmonary toxicity cases respond to cessation of amiodarone and initiation of steroid treatment, deaths can occur. The mortality was reported to be 10 to 23% in the literature [20, 21]. While there are different patterns of amiodarone pulmonary toxicity, different subtypes correspond to different prognosis. Acute respiratory distress syndrome (ARDS) due to amiodarone pulmonary toxicity is associated with mortality up to 50% [14, 20]. On the contrary, patients who presented with pulmonary nodules were reported to have better prognosis [16].

The studies on amiodarone pulmonary toxicity have significant heterogeneity with variable results on incidence and risk factors. As amiodarone pulmonary toxicity is a potentially fatal adverse effect from amiodarone use, identifying the risk factors for amiodarone pulmonary toxicity would enable clinicians to identify at-risk group and consider appropriate alternative anti-arrhythmic, and also timely arrange assessment imaging for patients on long-term amiodarone.

## Materials and methods

A multi-centre retrospective cohort study to identify the key clinico-epidemiologic factors that predict development of amiodarone pulmonary toxicity was conducted. List of patients on amiodarone from 1st January 2005 to 31st December 2015 was obtained from the Pharmacy Departments in three major regional public hospitals in Hong Kong (Queen Mary Hospital, Grantham Hospital and Queen Elizabeth Hospital). Queen Mary Hospital and Queen Elizabeth Hospital are two major regional acute hospitals and tertiary cardiac referral centres in Hong Kong, serving the Hong Kong West and Kowloon Central clusters respectively. Grantham Hospital is the only heart transplant centre in Hong Kong receiving cardiac referrals from the whole territory. There are 414, 630 and 282 medical beds (including coronary care units) in Queen Mary Hospital, Queen Elizabeth Hospital and Grantham Hospital respectively. All three hospitals provide in-patient and out-patient specialized cardiac services to patients with a variety of cardiac conditions. Chinese patients aged 18 years or above who had been on amiodarone for at least 90 days were included in the study in order to identify the development of chronic amiodarone pulmonary toxicity. Patients who had underlying interstitial lung disease prior to amiodarone exposure were excluded from the study as the development of amiodarone pulmonary toxicity among patients with underlying interstitial lung disease would be difficult to diagnose. The worsening of clinical and radiological features could be due to deterioration or exacerbation of the underlying interstitial lung disease or amiodarone pulmonary toxicity. In general, amiodarone would be avoided in patients with amiodarone pulmonary toxicity if possible.

Demographic data (age, gender, smoking status), relevant respiratory symptoms (cough, sputum, shortness of breath) and clinical data/investigations (underlying cardiac diagnosis, underlying arrhythmia, comorbidities, duration of amiodarone treatment, dose of amiodarone, complications during amiodarone treatment, blood tests for organ functions) were collected through clinical record review. The relevant chest X-ray and computed tomography (CT) films were reviewed to confirm the radiological findings and diagnosis. Ever-smoker was defined as one who had smoked at least one cigarette a day, pipe, water pipes, cigars, and hand rolled cigarettes, for one year or more. For patients who had received amiodarone treatment, the cumulative dose and duration of exposure were retrieved from review of clinical records. The renal function was assessed by estimated glomerular filtration rate (eGFR) by the Modification of Diet in Renal Disease (MDRD) Study equation.

 This study was approved by the Institutional Review Board of the University of Hong Kong/Hospital Authority Hong Kong West Cluster and Kowloon Central/East Cluster (HKU/HA HKW IRB approval no.: UW16-353, UW17-450; KCC/ KCC/KEC IRB approval no.: REC (KC/KE)-17-0237/ER-1).

The primary outcome was the development of amiodarone lung toxicity. The diagnosis of amiodarone lung toxicity was based upon all of the following criteria including new onset of pulmonary symptoms and/or new chest radiographic abnormalities without evidence supporting congestive heart failure, infectious processes or malignancy. All cases of amiodarone pulmonary toxicity were reviewed by investigators (WC Kwok and JCM Ho) to confirm the diagnosis. The final categorization of patients exposed to amiodarone into those with or without amiodarone pulmonary toxicity was determined by a consensus meeting of two investigators (WC Kwok and JCM Ho). In the consensus meeting, the patient’s clinical history, relevant imaging, laboratory test results and any treatment of amiodarone pulmonary toxicity were reviewed. Those who fulfilled the aforementioned criteria were classified to have amiodarone pulmonary toxicity.

### Statistical analysis

The demographic and clinical data are described in actual frequency or mean +/- SD. Baseline demographic and clinical data are compared between two outcome groups (with or without amiodarone pulmonary toxicity) by two sample proportion z test. Penalized logistic regression was used in the statistical analysis as the outcome is rare, providing bias-reduction for small sample size as well as yielding finite and consistent estimates even in case of separation [22, 23]. Multiple penalized logistic regression modeling is used to take into account of potential confounders including age, gender and smoking history of the subjects. Statistical significance is determined at the level of p = 0.05. To develop a predictive rule, variables univariately associated (p < 0.05) with development of amiodarone pulmonary toxicity were dichotomised or categorised and retained for multivariate testing. Cut-off points were identified using the following hierarchy: visual inspection of the receiver operator characteristic (ROC) curve; a clinically relevant cut-off; or a median split. Eligible variables were regressed against incidence of amiodarone pulmonary toxicity using backward stepwise methodology. Goodness-of-fit was assessed by Hosmer–Lemeshow statistic [21] and analysis of studentised residuals and leverage values identified and evaluated outliers. All the statistical analyses are done using version 4.0.5 of R: The R Project for Statistical Computing.

## Results

A total of 1786 patients on amiodarone from 2005 to 2015 that fulfilled the eligibility criteria were included in the study, with 8 patients with underlying interstitial lung diseases being excluded. Among the 1786 patients included, with 62.0% being male, mean age of 73.6 +/- 14.0 years, and 28.8% current or ex-smokers. Ischaemic heart disease was the most common (40.7%) underlying cardiac diagnosis, while supraventricular arrhythmia, especially atrial fibrillation, was the most common (71.3%) indication for amiodarone. For those with underlying lung diseases, chronic obstructive pulmonary disease was the most common, occurring in 112 patients (6.27% of the whole study population). There were 34 patients who had amiodarone pulmonary toxicity, accounting for an incidence of 1.90%, while 267 (14.9%) had amiodarone related thyroid dysfunction.

Among the 34 patients with amiodarone pulmonary toxicity, the mean age was 78.6 years, 22 (64.7%) were males and 15 (44.1%) were current or ex-smokers. Equal number of patients was taking amiodarone for supraventricular arrhythmia and ventricular arrhythmias. For the underling cardiac diagnosis, 7 (20.6%) patients had atrial fibrillation/supraventricular tachycardia alone, while 17 (50.0%), 1 (2.9%), and 6 (17.7%) had ischemic heart disease, chronic rheumatic heart disease and cardiomyopathy respectively. Eight (23.5%) patients had underlying lung diseases, including 1 (2.9%), 1 (2.9%), and 6 (17.6%) with asthma, bronchiectasis and COPD respectively. The mean estimated glomerular filtration rate (eGFR) was 60.8 ml/min/1.73m^2^ by the MDRD Study equation. Seven patients (20%) had amiodarone related thyroid dysfunction, with 2 (5.9%) thyrotoxicosis and 5 (14.7%) hypothyroidism. The demographics of the included patients are summarized in Table 1. The median time to develop amiodarone pulmonary toxicity was 66.8 months (Interquartile range = 74.5).

**Table 1:** Baseline demographic and clinical characteristics of 1786 patients treated with amiodarone

	Mean ± SD or number (%)		*p* value
	With amiodarone pulmonary toxicity	Without amiodarone pulmonary toxicity	
Gender, male	22 (64.7%)	1085 (61.9%)	0.741
Age, years	78.6 ± 11.7	73.6 ± 14.1	0.110
Current or ex-smoker	15 (44.1%)	500 (28.5%)	**0.047**
Cardiac diagnosis			0.231
Arrhythmia alone	7 (20.6%)	462 (26.4%)	0.308
Ischemic heart disease	17 (50.0%)	710 (40.5%)	0.420
Chronic rheumatic heart disease	1 (2.9%)	249 (14.2%)	0.183
Cardiomyopathy	6 (17.6%)	247 (14.1%)	0.397
Miscellaneous	3 (8.8%)	84 (4.8%)	0.207
Underlying arrhythmia			
Supraventricular arrhythmia	17 (50.0%)	1259 (71.9%)	0.005
Ventricular arrhythmia	17 (50.0%)	491 (28.1%)	0.009
Underlying lung disease			0.071
Asthma	1 (2.9%)	40 (2.3%)	0.683
Bronchiectasis	1 (2.9%)	23 (1.3%)	0.332
Chronic obstructive pulmonary disease	6 (17.6%)	106 (6.1%)	**0.005**
Miscellaneous	0 (0%)	3 (0.2%)	0.824
eGFR, ml/min/1.73m^2^	60.8 ± 24.7	64.4 ± 32.1	0.303
Duration of amiodarone, months	70.5 ± 43.1	46.1 ± 54.3	**0.019**
Daily amiodarone dose, mg	170.6 ± 52.4	165.3 ± 70.2	0.252
Cumulative dose, gram	356 ± 246	217 ± 306	0.421
Amiodarone related thyroid dysfunction			0.535
Thyrotoxicosis	2 (5.9%)	100 (5.7%)	0.326
Hypothyroidism	5 (14.7%)	160 (9.1%)	0.910

Bold indicates factors that are statistically significant

Older age, ventricular arrhythmia, underlying lung disease, longer duration of amiodarone use and higher cumulative dose of amiodarone were identified risk factors for development of amiodarone pulmonary toxicity. In univariate penalized logistic regression, the odds ratios (ORs) for age, ventricular arrhythmia, cumulative dose of amiodarone, duration of amiodarone use and underlying lung disease were 1.032 (95% confidence interval CI 1.002–1.063; *p* = 0.039), 2.564 (95% CI 1.299–5.063; *p* = 0.007), 4.556 (95% CI 1.781–11.653; *p* = 0.002), 1.010 (95% CI 1.004–1.016; *p* = 0.002), and 3.779 (95% CI 1.778–8.033; *p* = 0.001) respectively.

Multivariable penalized logistic regression was performed with toxicity adjusted for age, gender and smoking history. The OR was 1.047 (95% CI 1.010–1.085; *p* = 0.013) for age, 2.511 (95% CI 1.146–5.501; *p* = 0.021) for ventricular arrhythmia, 2.703 (95% CI 1.053–6.935; *p* = 0.039) for underlying lung disease and 4.762 (95% CI 1.310–17.309; *p* = 0.018) for cumulative dose of amiodarone.

Details of the statistical analysis of risk factors for amiodarone pulmonary toxicity are shown in Table 2.

**Table 2:** Penalized logistic regression model to predict amiodarone pulmonary toxicity

Variables	Univariate analysis Odds ratios (95% CI)	*p* value	Multivariable analysis Odds ratios (95% CI)	*p* value
Gender, male	0.887 (0.436–1.805)	0.741		
Age, years*	1.032 (1.002–1.063)	**0.039**	1.047 (1.010–1.085)	**0.013**
Current or ex-smoker	1.977 (0.997–3.921)	0.051		
Underlying lung disease*	3.779 (1.778–8.033)	**0.001**	2.703 (1.053–6.935)	**0.039**
Ventricular arrhythmia*	2.564 (1.299–5.063)	**0.007**	2.511 (1.146–5.501)	**0.021**
eGFR	0.996 (0.985–1.008)	0507		
Duration of amiodarone use	1.010 (1.004–1.016)	**0.002**		
Daily amiodarone dose	1.001 (0.997–1.005)	0.662		
Amiodarone-related thyroid dysfunction	1.481 (0.638–3.436)	0.360		
Cumulative dose of amiodarone*	4.556 (1.781–11.653)	**0.002**	4.762 (1.310-17.309)	**0.018**

Bold indicates factors that are statistically significant

*Factors that are statistically significant after adjustment for age, gender and smoking history

**Fig. 1:**
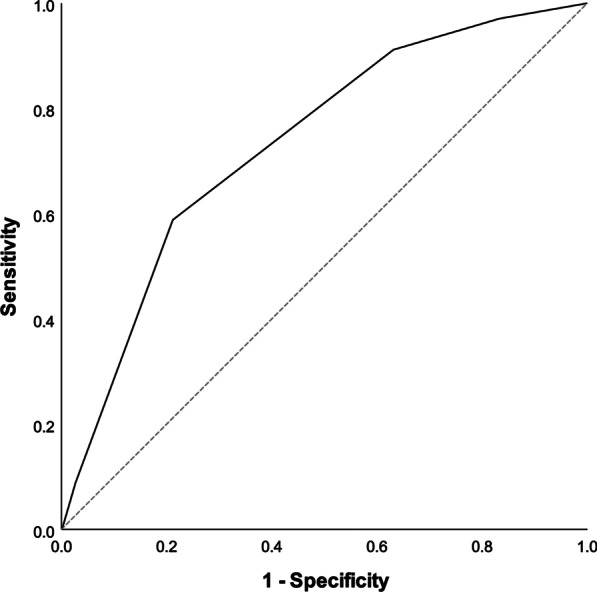
Receiver Operating Curve (ROC) for the proposed prediction rule for amiodarone pulmonary toxicity

**Fig. 2:**
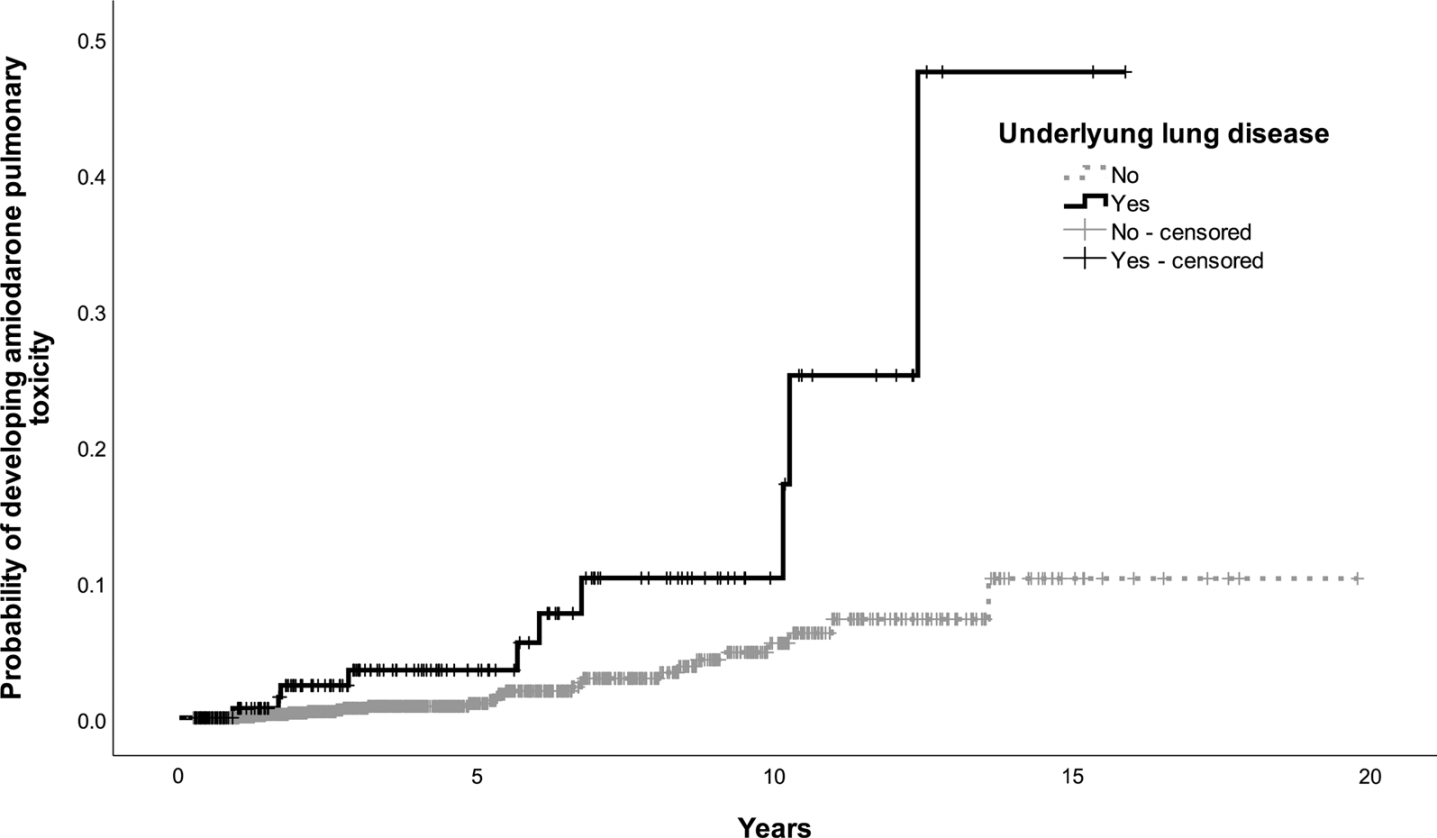
Kaplan–Meier curve for developing amiodarone pulmonary toxicity by age group

**Fig. 3:**
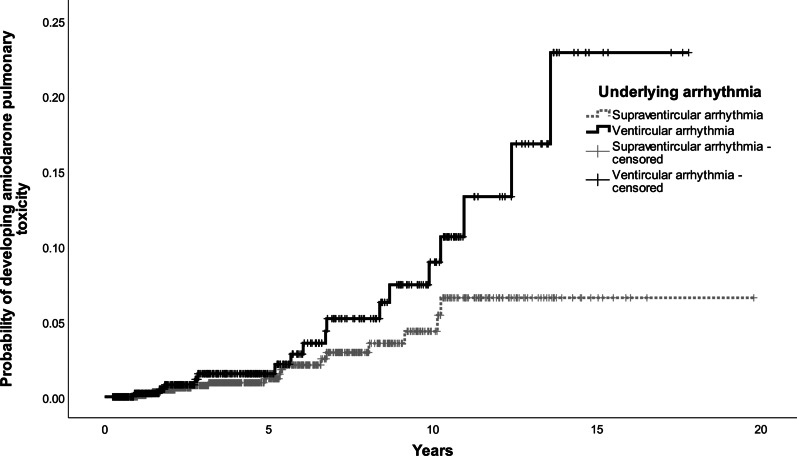
Kaplan–Meier curve for developing amiodarone pulmonary toxicity by underlying lung disease

**Fig. 4:**
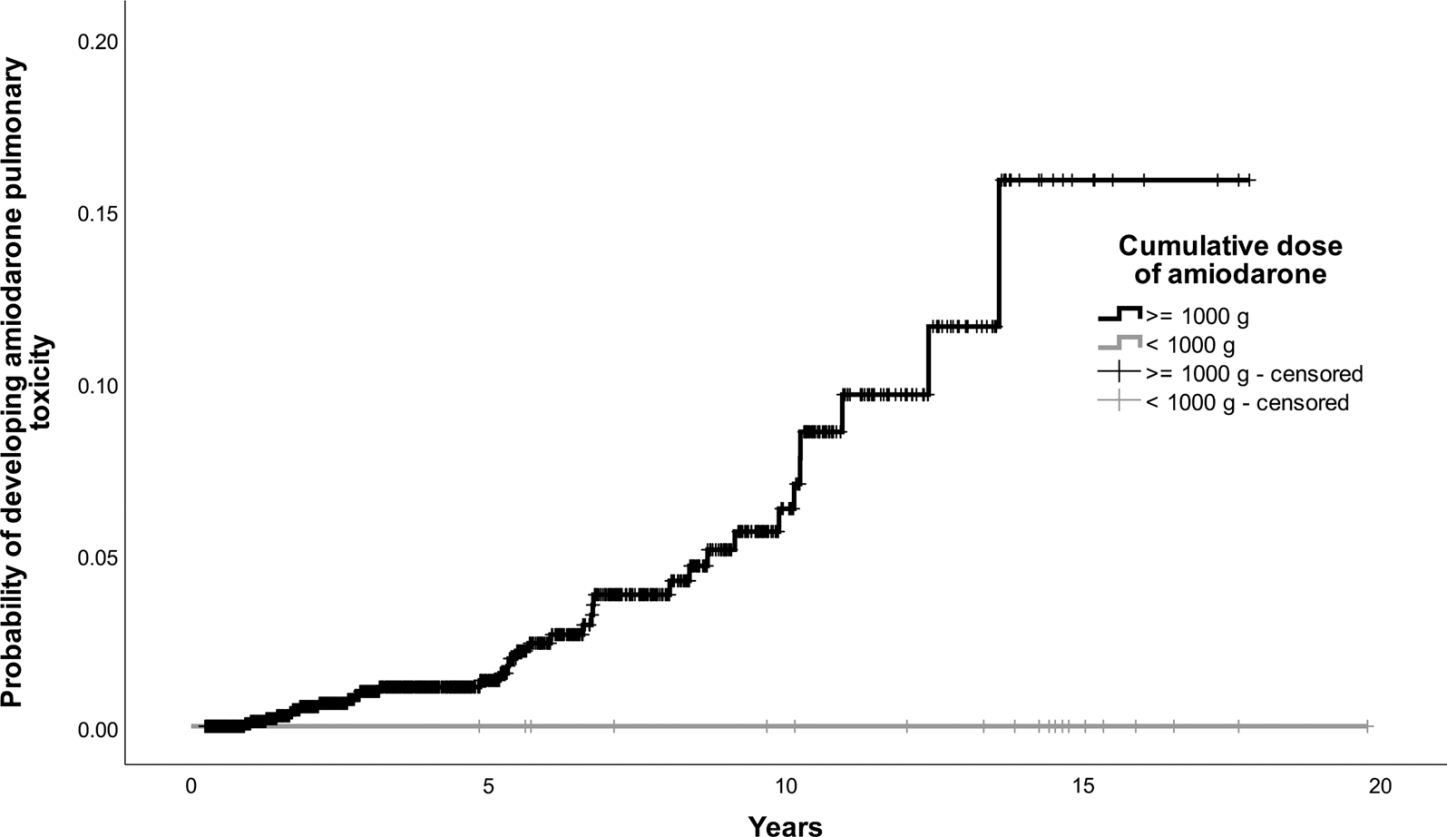
Kaplan–Meier curve for developing amiodarone pulmonary toxicity by underlying arrhythmia

**Fig. 5:**
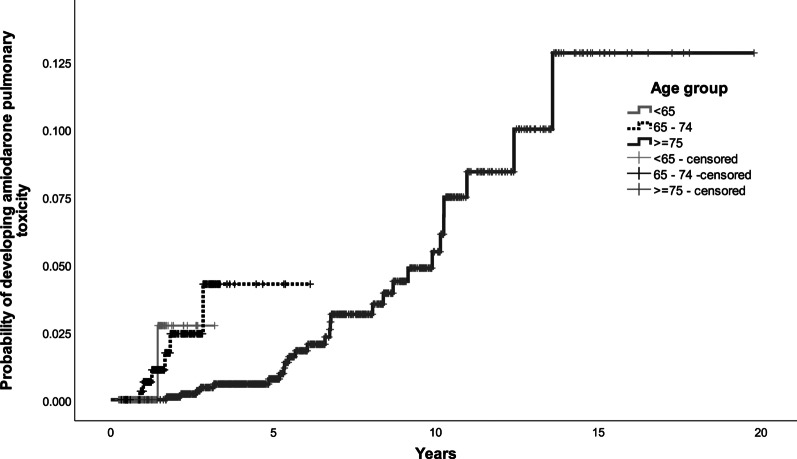
Kaplan–Meier curve for developing amiodarone pulmonary toxicity by cumulative dose of amidarone

Further work was done to categorize patients into risk groups, based on their risk of developing amiodarone pulmonary toxicity. Covariates with *p* < 0.05 (age with cut-off at 65 and 75, underlying arrhythmia, underlying lung disease and cumulative dose of amiodarone at cut-off at 1000 g) were included in the multivariate model to build the scoring system. One or two points were assigned to each risk factor and a five-point risk factor score is developed. Patients with 3 points or more were at increased risk of developing amiodarone pulmonary toxicity. At receiver operating characteristic curve (ROC) analysis, this score that defined high-risk group had area under the curve (AUC) of 0.733 (95% CI 0.652–0.813, *p* value < 0.001) (Fig. 1). The final prognostic score was the sum of individual scores, which were assigned with reference to the regression coefficients of the model. The scoring system is listed in Tables 3 and 4. The Kaplan-Meier curves for the four risk factors were shown in Figs. 2, 3, 4 and 5.

**Table 3:** Prediction score for development of amiodarone pulmonary toxicity

Risk factors	Score
Age (years)	
< 65	0
65–74	1
≥ 75	2
Underlying arrhythmia	
Supra-ventricular arrhythmia	0
Ventricular arrhythmia	1
Underlying lung disease	
No	0
Yes	1
Cumulative dose of amiodarone (g)	
< 1000	0
≥ 1000	1

**Table 4:** Probability of developing amiodarone pulmonary toxicity by the prediction rule

Score	Probability (%)
0	0.3
1	0.6
2	1.5
3	5.0
≥ 4	6.3

## Discussion

To our best knowledge, the current study is one of the largest scale studies on amiodarone pulmonary toxicity in an Asian population. A total of 34 cases with amiodarone pulmonary toxicity were identified among 1786 patients taking amiodarone from 2005 to 2015, accounting for an estimated incidence of 1.9%. The following factors were also identified to be significantly associated with amiodarone pulmonary toxicity, namely age, ventricular arrhythmia, cumulative dose of amiodarone and underlying lung disease. Apart from identifying the risk factors of amiodarone pulmonary toxicity, we also developed a five-point prediction rule based on the findings from multivariable penalized logistic regression. From the prediction rule, patients with 3 or more points are at increased risk of developing amiodarone pulmonary toxicity. Those with 3 and 4 points had 5.0% and 6.3% risk of developing amiodarone pulmonary toxicity respectively. This easy to use prediction rule may help clinicians to identify patients at risk of developing amiodarone pulmonary toxicity and consider early switch to an alternative anti-arrhythmic when appropriate.

Age has been shown to be associated with amiodarone pulmonary toxicity in the literature, with hazard ratio of 1.01 per year for patients above the age of 60 years [24]. Elderly patients are likely to have more co-morbidities and lower pulmonary reserve, which predispose them to the development of clinically evident amiodarone pulmonary toxicity. The relationship between underlying arrhythmia and amiodarone pulmonary toxicity can be related to the indication of amiodarone. In this study, ventricular arrhythmia is found to be significantly associated with amiodarone pulmonary toxicity, which is still valid in multivariable penalized logistic regression after adjusting for age, gender and smoking history. Ventricular arrhythmia is in general more dangerous than supraventricular arrhythmia with limited choice of anti-arrhythmic. We examined the characteristics of patients with supraventricular and ventricular arrhythmia. The age, daily amiodarone dose, duration of amiodarone and cumulative amiodarone dose were similar in both groups. This suggests that the observed association cannot be explained by the dose or duration of amiodarone exposure. The exact reason for this observation remains elusive.

The issue on dose of amiodarone and its toxicity has been assessed in prior studies. Cumulative dose of amiodarone has been shown to be associated with toxicity and this is again demonstrated in the current study. The development of amiodarone pulmonary toxicity long after exposure (months to years) is also consistent with cumulative toxic effects. In our study, lower dose amiodarone (less than 200 mg daily) can also lead to amiodarone pulmonary toxicity, suggesting that the cumulative effect with prolonged exposure is the key risk factor.

Underlying lung disease has been reported to be associated with amiodarone pulmonary toxicity and is also shown in the current study, though there is conflicting evidence in the literature. In the Atrial Fibrillation Follow-up Investigation of Rhythm Management (AFFIRM) study, preexisting pulmonary disease was shown to be associated with a higher risk of diagnosed amiodarone pulmonary toxicity [25]. Nonetheless, use of amiodarone in the presence of preexisting pulmonary disease did not increase pulmonary death and all-cause mortality rate. On the contrary, the Congestive Heart Failure-Survival Trial of Antiarrhythmic Therapy (CHF-STAT) study demonstrated no accelerated loss of diffusing capacity for carbon monoxide (DLCO) among patients with COPD who received amiodarone, compared with those who received placebo [26]. However the CHF-STAT study only had 519 subjects and 269 of them were taking amiodarone. With the low incidence of amiodarone pulmonary toxicity (as low as 1% in some studies), the relatively small sample size in the CHF-STAT study did not provide sufficient statistical power in identifying this association. There is also some criticism that patients with limited pulmonary reserve become symptomatic earlier in their course than other individuals, which makes underlying lung disease apparently a risk factor of amiodarone pulmonary toxicity.

There are also discrepancies from the literature regarding other risk factors of amiodarone pulmonary toxicity. In this study, gender was not found to be significantly associated with amiodarone pulmonary toxicity. Jackevicius et al. reported that male gender was associated with amiodarone pulmonary toxicity [3]. But male patients are more likely to have underlying COPD and the relationship between gender and amiodarone pulmonary toxicity may partly be explained and confounded by underlying COPD in the male patients. Male patients may also have ischemic heart disease with subsequent ventricular arrhythmia. The association between amiodarone pulmonary toxicity and gender may be medicated by the co-morbidities instead of gender itself. Regarding the association between underlying renal disease and amiodarone pulmonary toxicity, it was shown to have a hazard ratio of 1.26 according to Jackevicius’s study [3]. But it is known that amiodarone is metabolized by the liver and excreted via hepatobiliary route. There is minimal elimination of both amiodarone and monodesethylamiodarone by the kidneys due to large volume of distribution and extensive protein binding. Thus dose adjustment is not necessary for patients with renal function impairment or even those receiving renal replacement therapy. Theoretically, underlying renal impairment does not affect the serum level of both amiodarone and monodesethylamiodarone, hence minimal impact on risk of amiodarone pulmonary toxicity. This is in line with the finding from the current study.

Although amiodarone is well-known to cause pulmonary toxicity and thyroid dysfunction, it remains one of the most commonly used anti-arrhythmics as it is available in both oral and parenteral route, with a wide range of indications. Other pharmacological and interventional treatment options are also available for supraventricular and ventricular arrhythmia, with different risks of adverse events.

There are several limitations of this study. First of all, only 34 patients with amiodarone pulmonary toxicity can be identified. The relatively small number of cases with amiodarone pulmonary toxicity limits the statistical analysis on potential risk factors, especially in performing subgroup analysis with even smaller sample size. Penalized logistic regression was therefore used to correct the bias of estimation due to rare occurrence of amiodarone pulmonary toxicity. Nonetheless, our study is one of the largest scale in Asia, which included 1786 patients with amiodarone exposure. A population-based study with much bigger sample size is needed to confirm our findings and validate the prediction rule for development of amiodarone pulmonary toxicity. The prediction rule that we developed may have the problem of overfitting as cross-validation was not conducted. While a prediction rule was developed, ideally using an independent cohort to validate the results could help to assess the performance of the rule. Internal validation techniques could be applied to correct for overfitting and optimism but the accuracy would still be substantially lower than using an independent cohort. It was proposed that when overfitting could not be adequately prevented or adjusted during development of the rule, all regression coefficients could be adjusted with a single correction factor estimated from the data of new patients in the validation set [27]. Recruiting an independent cohort to validate the prediction rule that we proposed would solve this problem. As a study that aim to examine the risks development of amiodarone pulmonary toxicity, the patients were identified by their record of amiodarone prescription. Patients taking some other drugs that can potentially cause pulmonary toxicity together with amiodarone were excluded in this study in order to avoid diagnostic uncertainty in these cases Serum monodesethylamiodarone level was not measured in this study given the retrospective design. The test is unavailable as a standard routine in the biochemistry laboratory in Hong Kong. Serum monodesethylamiodarone level has been shown to be associated with amiodarone pulmonary toxicity and is worth assessing in a prospective study setting. The cumulative dose of amiodarone which has been investigated in this study may serve as a surrogate when the serum monodesethylamiodarone level is not available, though the serum monodesethylamiodarone level can be affected by liver function impairment and CYP3A4 genetic polymorphism apart from the cumulative dose of amiodarone.

## Conclusions

The incidence of amiodarone pulmonary toxicity among Chinese patients in Hong Kong is estimated to be 1.9% in this study. Age, underlying lung disease, ventricular arrhythmia and cumulative dose of amiodarone are associated with the development of amiodarone pulmonary toxicity. A simple prediction rule is developed to risk stratify patients with amiodarone exposure.

.

## Data Availability

All data generated or analysed during this study are included in this published article.

## Availability of data and materials

All data generated or analyzed during this study are included in this published article [and its supplementary information files].
